# Machine learning-based ABA treatment recommendation and personalization for autism spectrum disorder: an exploratory study

**DOI:** 10.1186/s40708-022-00164-6

**Published:** 2022-07-25

**Authors:** Manu Kohli, Arpan Kumar Kar, Anjali Bangalore, Prathosh AP

**Affiliations:** 1 Indian Institute of Technology-Delhi, Department of Management Studies, IV Floor, Vishwakarma Bhavan, Shaheed Jeet Singh Marg, Hauz Khas, New Delhi, 110016 India; 2 ICON Centre, K. M. Chavan chawk, Shivajinagar Road, Garkheda, Aurangabad, 431005 India; 3 Indian Institute of Science, CV Raman Rd, Bengaluru, 560012 Karnataka India

**Keywords:** ABA, ASD, Autism, Collaborative filtering, Machine learning, Patient similarity

## Abstract

Autism spectrum is a brain development condition that impairs an individual’s capacity to communicate socially and manifests through strict routines and obsessive–compulsive behavior. Applied behavior analysis (ABA) is the gold-standard treatment for autism spectrum disorder (ASD). However, as the number of ASD cases increases, there is a substantial shortage of licensed ABA practitioners, limiting the timely formulation, revision, and implementation of treatment plans and goals. Additionally, the subjectivity of the clinician and a lack of data-driven decision-making affect treatment quality. We address these obstacles by applying two machine learning algorithms to recommend and personalize ABA treatment goals for 29 study participants with ASD. The patient similarity and collaborative filtering methods predicted ABA treatment with an average accuracy of 81–84%, with a normalized discounted cumulative gain of 79–81% (NDCG) compared to clinician-prepared ABA treatment recommendations. Additionally, we assess the two models’ treatment efficacy (TE) by measuring the percentage of recommended treatment goals mastered by the study participants. The proposed treatment recommendation and personalization strategy are generalizable to other intervention methods in addition to ABA and for other brain disorders. This study was registered as a clinical trial on November 5, 2020 with trial registration number CTRI/2020/11/028933.

## Introduction

Autism spectrum disorders (ASD) prevalence in the US (United States) is estimated at 1 in 44 children [1], a rise from previous figures of 1 in 54. Given the brain’s high neuroplasticity in the first 5 years [2], gold-standard ABA intervention [3] can improve the skills of children with ASD enhancing their language, life skills [4, 5], and IQ (intelligence quotient) [6]. ABA interventions have demonstrated impactful outcomes for a wide range of children with ASD and also with other brain disorders such as ADHD [7], cerebral palsy [8] in multicultural environment [9]. Intensive ABA intervention can improve challenges related to adaptive behavior, ASD severity, and academic performance [10–12]. Additionally, ABA therapies in low-resource countries have enhanced skill acquisition and inclusion possibilities [13] for ASD children. However, there is an acute shortage of certified ABA professionals [14, 15], doctors specializing in child and adolescent brain disorders and clinicians practicing evidence-based intervention methods such as EIBI [10], occupational therapy [16], and speech therapy [17]. The shortage of clinicians impacts the access, reach, and affordability of treatment services, especially in low-resource settings, where approximately 80% of children diagnosed with ASD live [18]. Further, the limited availability of licensed ABA clinicians impacts the quality of the baseline ABA program and its subsequent revisions during the patient treatment journey [19] limiting rehabilitation outcomes for ASD children. The above limitations underscore the importance of technological advancements, particularly in developing technology-driven personalized ABA recommendation systems (RS), enhancing therapist capacity [20, 21] and intervention quality, disencumbering both caregivers and healthcare professionals of the challenges they face. Additionally, there is growing interest in applying ML algorithms to the field of brain disorders and mental health [22, 23]. The application of ML on the large clinical data [24] can ensure trustworthy and efficient healthcare decisions benefiting both patients [25, 26] and providers [27]. Personalization facilitated by ML has become engrained in our daily interactions with a variety of digital systems [28], including e-commerce [29], movie recommendations, exercise advice [30], and therapy recommendations [31]. Thus, personalized RS in ASD management can utilize patients’ medical meta-data and assessment records to recommend personalized treatment prescriptions to improve their well-being that would not be achievable through conventional procedures [32]. Due to the established efficacy of RS and a desire to overcome the limitations of the traditional ABA intervention model, we conducted a first-of-its-kind pilot study evaluating the effectiveness of RS with treatment personalization capabilities. We used assessment records, sociodemographic data such as age and gender [33, 34], and 6-month longitudinal treatment data from 29 ASD children.The paper is organized with a Literature review in Sect. 2, followed by a Materials and methods in Sect. 3. We then present Results in Sect. 4 followed by Discussion and conclusion in Sects. 5 and 6, respectively.

## Literature survey

This section highlights literature on the related work with the following subsections. Sect. 2.1 highlights various assessment scales used in the assessment of brain disorders. Sect. 2.2 highlights the role of technology in ASD management. In addition, Sect. 2.3 discusses the use of patient similarity models on multimodal clinical data. The Sects. 2.4 and 2.5 discuss ML-based treatment recommendation and personalization methods.

### Brain disorders and assessment scales

Numerous evaluation techniques, including structural and functional neuroimaging [35], brain electrophysiology [36], molecular genetics [37], and clinical assessments [38, 39] across cognitive and behavioral dimensions, are crucial for improving diagnostic precision for various brain disorders. fMRI is the non-invasive technique for assessing the functioning of various brain regions responsible for critical functions such as thought, speech, movement, and sensation. The technology analyzes functional brain anatomy, assesses the effects of stroke, stress, or retrogressive disease (such as osteoporosis or cancer) on brain function, and examines the progression and function of brain tumors [40, 41]. Additionally, the approach aids in planning invasive therapies such as brain surgery. An EEG records brain wave patterns that can assist a physician in identifying aberrant patterns indicative of seizures and other difficulties with brain function. EEGs can be used to diagnose problems such as sleep disorders [42] and behavioral disturbances and analyze brain activity following a severe head injury. The researchers have used genetic testing to determine whether the person inherited one of the known genes associated with the brain disorder [40]. Additionally, clinical testing can find mutations in specific genes or sets of genes to establish a specific brain disorder diagnosis or provide information to clinicians to make treatment recommendations. Behavioral assessments on the physiological, cognitive, motor, speech or socio-communication components of behavior can facilitate diagnosis, severity, and treatment design for various brain disorders. These assessments increasingly use validated rating scales to document and record patient responses against a set of questions or record participant behavior responses against activities or tasks. These assessment scales have been deployed to make diagnoses and treatment roadmap for conditions such as Parkinson [43], Alzheimer [44], multiple sclerosis [45], dementia in the elderly [46], ADHD [47], and ASD [48, 49].

### Role of technology in ASD management

Modern medicine faces difficulty utilizing the extensive knowledge base required to diagnose and treat complicated mental health issues. With the abundance availability of structured and unstructured data, ML is increasingly deployed to manage multiple mental health conditions [27], such as epilepsy [50], and Alzheimer [51]. ML is a collection of algorithms that infer meaningful patterns from data without requiring human intervention [52]. The goal of ML is to replicate human cognitive functions. The ML application brings a paradigm shift in the healthcare sector with early diagnosis [51], personalized treatment [53], and drug discovery [54] by analyzing extensive data, improving access and quality of services to manage multiple health conditions. Recently, there has been a rise in studies involving ML in managing mental health conditions [55]. For example [56], developed a solution to promote adherence to the consumption of drugs to manage conditions such as schizophrenia. Ref. [57] identified persons at CHR of developing psychosis using web-based risk screening. ML can be used to detect depression by identifying putative fMRI biomarkers of vulnerability to major depression [58]. The application of technology-focused solutions has played an essential role in managing ASD. The application of DL technologies to diagnosing brain disorders is emerging as a new area of research. Ref. [59] generated virtual brain networks using fMRI data and developed a unique CNN to diagnose ASD. Ref. [60] tracked the eye movements of individuals with and without ASD while they browsed web pages. They trained machine learning classifiers on visual processing data patterns and predicted ASD 74% accurately. Further [61], used ML models to build a behavior-based automated screening using video and audio data to identify 8–24 months HR-ASD infants. Typically, children with ASD have poor IJA skills. Young children usually perceive IJA through nonverbal gestures such as pointing, sharing, showing, and collective gaze [62]. Ref. [63] developed an immersive C3I platform to assist youngsters with ASD to practice IJA skills. The platform incorporates a caregiver into the instructional loop, retaining the benefits of both human-administered and computer-administered intervention. Further, socially assistive robots may aid in treating ASD by training social skills through games that utilize dyadic interactions. Ref. [64] created a robotic coaching platform to improve the social, physical, and cognitive skills of ASD children.

### Patient similarity

The traditional one-size-fits-all clinician-centric decision model has evolved to a data-driven predictive framework [65]. The new paradigm incorporates patient-centric tailored disease onset risk computation, treatment prediction, dosage recommendations, and treatment revisions depending on disease severity, progression, and symptoms [66, 67]. Several machine learning-based patient similarity models are constructed based on multimodal data that capture disease onset, severity, symptomology and track disease evolution with various treatment combinations [68]. An individualized treatment or diagnostic framework includes methods to compute the similarity between a new patient and an existing large pool of patients [69] in the EMR. Several distance metrics such as Euclidean, Mahalanobis, and cosine are computed using the patient’s sociodemographic and clinical evaluation meta-data to derive patient similarity scores [70]. Most patient similarity models incorporate clinician-recommended disease-specific features that can be assigned weights [71] according to their importance. Further, by mapping disease subtypes [72] to an individual patient’s risk exposure [73], the patient similarity framework has resulted in the CDS framework [74] for early risk identification. For example, to predict the onset of diabetes, [71] shortlisted clinically relevant features, identified similar patients using LSML from the cohort, calculated risk score, and individualized risk profile for a new patient. Additionally, patient subgroups that may benefit from one treatment over another can be identified, establishing the efficacy and personalization of drugs and therapies [75] for a patient. Additionally, time series and clustering [68] are two techniques for identifying comparable patients based on meta-data from temporal clinical evaluations. The clustering method generates patient groups [53, 76] with comparable disease progression and clinical data patterns to predict whether a new patient will belong to the most similar cluster. For example, adult spinal deformity patients were classified using hierarchical clustering [77] to help surgeons optimize treatment and identify the least risky surgical choices. Further [78], constructed a two-dimensional RNN that learns patient similarity from longitudinal and multimodal data and improves recommendations and outcomes for Parkinson’s intervention. One of the difficulties of deploying supervised learning techniques is the time and expense associated with data labeling. [79] overcame the limitation by employing a weak supervision method. Cancer patients’ disease-subgroup classification was performed using supervised learning techniques and then integrated with unsupervised learning methods as a patient similarity vector. This resulted in several cluster groups useful for a precision treatment analysis.

### Treatment recommendation

Previous treatment recommendation systems classified diseases and medications using expert systems, supporting physicians in making more informed clinical judgments. However, with the introduction of EMRs [69] and the availability of a large amount of clinical data, real-time data-driven treatment guidance is finding prevalence [80]. Finding historical records of similar patients may aid in finding comparable reference cases for anticipating clinical outcomes and may provide a mechanism for heterogeneous label propagation to shortlist effective drugs and treatment regimens for a new patient. The primary data-driven treatment recommendation research methodologies are supervised learning (SL) and reinforcement learning. The goal of SL for prescriptions is to bridge the gap between the algorithm’s suggested medications and those advised by physicians. Numerous pattern-based algorithms [75] create prescription suggestions based on patient similarity and improve recommendation outcomes by learning associations between several diseases and multiple medication categories [81]. Precision treatment in mental health is a promising technique to boost psychotherapy’s efficacy. The therapy recommendations could be incorporated into a comprehensive treatment navigator to assist clinicians in making more informed clinical judgments and improving patient outcomes [82]. Unfortunately, there are two challenges with SL-based model recommendations. The first challenge is establishing the empirical basis for a "good” treatment plan defined by the medical literature. Second, the clinical decision system’s primary objective was to enhance patient outcomes, rather than matching prescriptions to a class label, usually ignored by the SL technique. Further, applying these methodologies to clinical practice presents difficulties due to their reliance on a small amount of data.

### Treatment personalization

The researchers are interested in establishing models that enable them to make tailored treatment suggestions. Without the supervisor’s oversight, the RS may prescribe markedly different medicines from those suggested by clinicians, offering unacceptable hazards [83]. These limitations can be managed using reinforcement learning for DTR [84] that can generate tailored treatment depending on a patient’s dynamic state over time. The appropriate DTR is obtained by optimizing the assessment signal to ensure long-term treatment effectiveness. For example [85], employed tabular Q-learning to make drug recommendations based on actual clinical data for schizophrenia patients. Collaborative filtering (CF), often used in an e-commerce platform, creates personalized recommendations based on user similarity by calculating a weighted average of user and item preference interactions [86, 87]. Most collaborative filtering systems use similarity indexes to assess the active user’s similarity to other users via a neighborhood-based method. Additionally, the CF models can automatically learn feature embedding rather than manual feature engineering. In a healthcare scenario, we can presume that individuals with comparable disease profiles or health concerns will receive similar treatments services in the healthcare domain based on a sparse, multi-dimension, and missing value utility matrix [88]. For example [89], used the CF technique to develop personalized recommendations to manage diabetic conditions. The patient’s age and vitals are analyzed to identify similar patients. Then using pre-treatment assessment and longitudinal treatment data, state-of-the-art supervised, collaborative, and content-filtering ML algorithms efficiently predict treatment goals, personalization, and effectiveness across time horizons.

## Material and methods

The study participant enrollment details are listed in Sect. 3.1, treatment planning and goal setting in Sect. 3.2 and study design in Sect. 3.3. We discuss the implementation of two ML algorithms for treatment recommendation and personalization in Sect. 3.4.

### Study detail and participants

The trial is based on a pre-post single-group design approved by the Indian Institute Of Technology, Delhi’s Ethics Committee, and registered with India’s clinical trial registry (CTRI/2020/11/028933). The study lasted from November 2020 to October 2021 and enrolled 31 ASD children aged 2 to 6 years diagnosed with standardized tools such as DSM-V [90], CARS-2 [91], ADOS [49], INDT-ASD [92], or ISAA [93]. The trial had three objectives: (1) to develop a behavioral treatment model using a digital platform that enhances parents’ and experts’ capacity to manage autism spectrum disorders better; (2) evaluate the efficacy of digitally delivered ABA intervention with parents as primary caregivers; and (3) to develop and validate machine learning models to recommend and personalize behavioral treatment plans using longitudinal treatment data of children across multiple domains, such as expressive language, receptive language, echoic, and requesting. This study covers the third objective of the trial. A social media campaign and referrals from developmental pediatricians and pediatric neurologists were used to recruit study participants. The study enrolled participants for 6 months and trained parents to play a caregiver role for their children by requiring them to attend weekly 1-h online consultations and training sessions with ABA therapists. The sociodemographic data, including age, gender, ethnicity, and ASD diagnosis for participants, were collected. From each family, informed consent was recorded. We did not include two parents for final enrollment due to our inability to confirm the child’s diagnosis. The entire study was conducted online. The final study consisted of 29 participants, including 24 males and five females, with a mean age of 4.12 years and a standard deviation of 0.94 years and age distribution listed in Table 1. The participants reported their ASD diagnosis from multiple diagnostic tools. Following were the participant inclusion criteria:

1. Children between the ages of 2 and 6 of both sexes.
2. Children should have a diagnosis of autism spectrum disorder using standardized instruments such as the DSM-V, CARS-2, ADI-R, INDT-ASD, ISAA, or any other evidence-based ASD diagnostic tool.
3. Children who are currently not undertaking ABA therapy.
4. The families should have access to any one of the devices, i.e., IOS or Android-based smartphones, desktops, or laptop.
5. The willingness of parents to participate in initial online training sessions and biweekly online training sessions with the therapists.

Following were the exclusion criteria:

1. Children with visual and aural impairments.
2. Children with a recent ailment, seizure history, or another chronic condition.
3. Young children with severe or profound GDD.
4. The child and his family do not speak English, Marathi, Bengali, or Hindi.
5. A history of traumatic brain injury or another significant medical or neurological disorder affecting motor or higher cortical function.
6. Severe intellectual disability or sensory–motor difficulties.
7. Caregivers or parents cannot use mobile, internet, desktops, or laptops to access remote training and evaluations.

**Table 1:** Enrollment details

Age group	Number of learners
Two–three years	4
Three–four years	8
Four–five years	12
Five–six years	5
Total	29

### Treatment planning and goal setting

The trained ABA therapists worked under the supervision of a BCBA and delivered parent training, performed goal setting and assessments, and updated the ABA program after discussing with the participant’s parents and family members during weekly meetings. The therapist prepared a comprehensive ABA program with written instructions and training videos and made it accessible via a mobile and web application. Parents used mobile or web applications to track their child’s progress, shared 10–15 min child’s progress videos weekly, and recorded responses to skill development treatment goals. At the start of months zero, four, and six, the children underwent a detailed SRS-2 and VB-MAPP assessment. We have detailed about SRS-2 [94] and VB-MAPP [95] assessment tools in Appendix 1.

### Study design

Personalized treatment goals for each child is a critical component of an ASD treatment strategy. The ABA therapist assessed each study participant’s skills at the start of the study using assessment tools such as the SRS-2 and VB-MAPP. These assessments suggested participants’ strengths and weaknesses and aided ABA therapists in developing a treatment plan tailored to each child. The therapist prioritized skills to include in the treatment plan based on the child’s age, level of functioning, areas of skill deficiency, family needs, and available time for family members to assume the caregiver role.

The ABA therapist chose the most appropriate skills from various domains to be part of the treatment plan, including social communication, social skills, academics, behavior management, and self-regulation. While each child’s needs are unique, ABA therapists frequently prioritize socio-communication skills to maximize skill development in core ASD deficit areas. However, a therapist must choose from a vast repertoire of skills in each domain to incorporate them into the treatment plan. As a result, ABA therapists’ and supervisors’ experiences and subjective judgments are often used to choose and prioritize skills to be part of the treatment goal.

ML models can overcome the subjectivity inherent in human-centered treatment selection through a data-driven approach. We evaluated the role of ML models in treatment recommendation and personalization using retrospective longitudinal treatment data of the study participants. We divided the treatment data into two parts: (1) domain and verbal operants, and (2) target codes:

- Level 1—Domains [96], and verbal operants [97] such as academic language, prerequisites, tacting, manding, expressive language, imitation, and receptive language lays down the foundation for developing language and communication skills. Skill development in various verbal operants is critical for language and communication development. Ref. [98] assert that developing skills within one verbal operant facilitates growth in other verbal operants.
- Level 2—Under each domain or verbal operant, a target code is a skill [99] expected to be learned by a participant. Usually, the targets under various domains are incorporated into the treatment goal in easy to complex chronological order.

We split each child’s retrospective treatment plan into a combination of a domain and target codes and implemented patient similarity and collaborative filtering ML models. We compare predictions made by two ML models to the treatment goal prepared by the ABA therapist, i.e., the ground truth. We assume that children would benefit from ABA treatment goals that have shown success to an existing child with similar gender, age, and assessment scores. We also compared the effectiveness of the recommended treatment from both ML models.

### Methods

We implemented and compared two ML methods for patient treatment recommendation and personalization:

1. Patient similarity with similar patient skill selection using cosine similarity method, and
2. Collaborative filtering.

### Cosine similarity

**Fig. 1:**
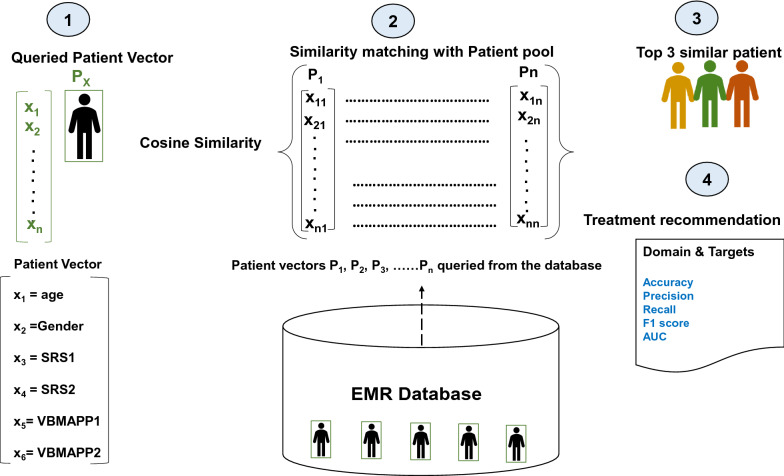
Patient similarity framework

The cosine similarity coefficient [100] quantifies the similarity of two vectors in an inner product space by computing the cosine of the angle between them and evaluating if the vectors point in the same direction. The coefficient is used in text analysis to determine the document’s similarity. When selecting a threshold for similarity, a value more than 0.5 [101] indicates strong similarities. We calculated cosine similarity and patient similarity metric (PSM) for 29 patients. PSM is defined as the similarity between two vectors of an inner product space:

$$\begin{aligned} {\text{PSM}}(P_1,P_2) = \frac{{}P_1 \cdot P_2}{\Vert P_1\Vert \Vert P_2 \Vert }, \end{aligned}$$

where $P_1$ and $P_2$ are predictor vectors corresponding to two distinct patients, respectively, where $P_1$ is the index patient and $P_2$ is the second patient, and finally, the pairwise patient similarity metric $PSM(P_1,P_2)$ is calculated. This study aimed to determine whether the patient cosine similarity score can aid treatment prediction and personalization. The therapist developed a treatment plan during the 6-month study duration at regular intervals. At various temporal points, we used the patient similarity framework to evaluate if treatment similarity concerning treatment commonality and effectiveness was observed in the study participants with similar patient similarity scores, as illustrated in Fig. 1 [102]. The figure details that a large number of patient records exist in EMR. Each patient record consists of sociodemographic, treatment, and assessment records. A new patient in the EMR is compared with the existing patient’s database using the cosine similarity Algorithm 1, on sociodemographic information and assessment records resulting in the recommendation of a similar patient cohort. The top three patients’ treatment records are suggested to a clinician as a treatment recommendation, from which a clinician can choose the optimal treatment option for the new patient.

We retrospectively analyzed the ABA program developed by the clinicians for 29 study participants. We calculated patient similarity by building vectors that included sub-module assessment T-scores of SRS-2 and aggregate scores of VB-MAPP along with age and gender. Thus, we evaluate each patient’s relevance to a skill that is inherently captured by the feature set and scores of SRS-2 and VB-MAPP. We compared individual patient treatment goals developed by clinicians with the top three similar patients as described in Algorithm 1 on commonality and effectiveness measures. Commonality refers to the percentage similarity of domains and targets in the treatment plan recommended by the ML model compared to the manual treatment plan developed by an ABA therapist (ground truth). Therefore, commonality measures prescription similarity, i.e., similar patients’ common domain and target codes. Observance of similar treatment for similar patients could lead the way for personalization. Effectiveness measures the percentage of ML-based recommended targets that the child mastered. We measured effectiveness at the end of months 3 and 6.
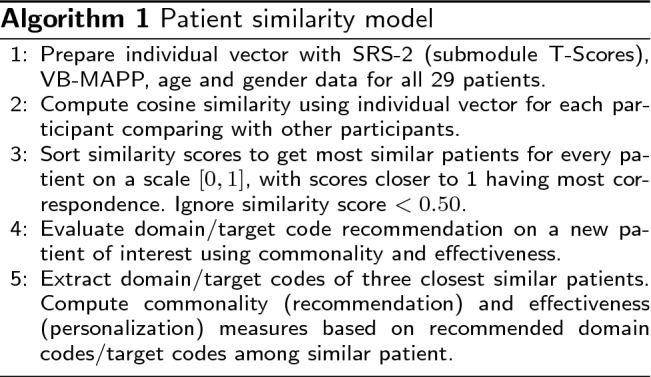

### Collaborative filtering

**Fig. 2:**
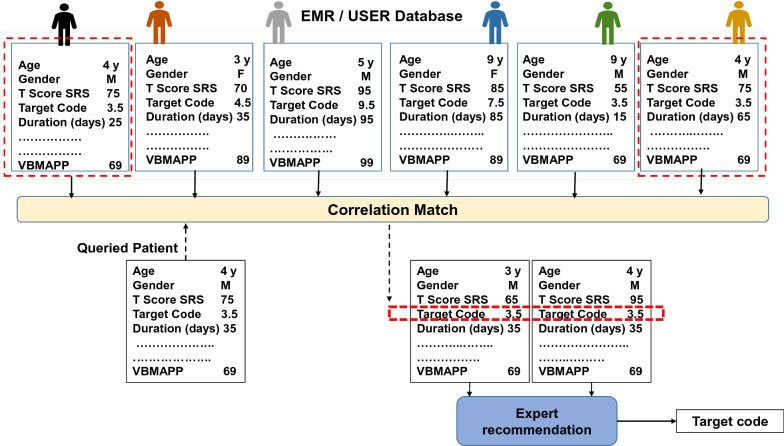
Collaborative filtering method for domain and target code recommendation

We used CF to recommend domain or target codes as part of the treatment plan for patients with similar clinical histories, treatment trajectories, and sociodemographic profiles. As shown in Fig. 2, the CF algorithm [103] incorporates demographic data (age and gender of patients), longitudinal treatment data and their effectiveness (number of days required to master a skill), and assessment data (SRS-2 and VB-MAPP).

The standard CF recommendation models can make treatment recommendations based on assessment and sociodemographic data available during the initial patient enrollment stage with inherent cold-start limitations. However, the algorithm can be integrated and optimized with treatment effectiveness data to generate recommendations and personalizations during the steady-state treatment phase, circumventing the inherent limitations.

We employed the CF-based algorithm recommendation model to create each patient’s user–item interactions with the measure we wish to forecast, i.e., treatment goals. Latent information regarding interactions, such as clicks, likes, dislikes, skips, views, and purchases, is frequently present in traditional e-commerce-based CF-based recommender systems. However, we observed that our use case lacked explicit rating data. Our data are implicit, meaning they do not include any external ratings, preferences, or votings provided by users on domain or treatment codes, an item of our recommendation interest. Nonetheless, our objective is to capture indirect rating information about user–item interactions as an implicit feedback system. Therefore, we investigated the relationship between patients’ treatment profiles, including sociodemographic data (age, gender), domain and target codes, treatment duration, and effectiveness (days to mastery) as interaction items and as an input vector to develop an effective treatment recommendation system using CF.

Using CF with implicit feedback alternating least square (ALS) technique [103], we can infer a preference/rating for each patient–target skill interaction that has occurred. Here, we select SRS-2 T-scores as a preference since it directly relates to the quantitative skill measurement criteria linked with domain and target code selection.

There are several ways to handle implicit feedback systems [104, 105]. We use ALS matrix factorization model approach [103] in the current study. We are interested to model the preference/rating ${\hat{r}}$ an user *u* would give to an item *i* by ${\hat{r}}_{ui} = x_u^{T}y_i$, where $x_u^{T} = (x_u^{1},x_u^{2},...,x_u^{N})$ is a vector associated with the user (patient), and $y_i^{T} = (y_i^{1},y_i^{2},...,y_i^{N})$ is a vector associated with the item (skill/target code). We define user vectors into a matrix

$$\begin{aligned}X^{T}= \begin{bmatrix} . &{} . &{} &{} .\\ . &{} . &{} ... &{} . \\ . &{} . &{} &{} . \\ x_{u_1} &{} x_{u_2} &{} ... &{} x_{u_{n_{{\text{users}}}}} \\ . &{} . &{} &{} . \\ . &{} . &{} ... &{} . \\ . &{} . &{} &{} . \end{bmatrix} \end{aligned}$$

and item vectors into a matrix

$$\begin{aligned}Y^{T}= \begin{bmatrix} . &{} . &{} &{} .\\ . &{} . &{} ... &{} . \\ . &{} . &{} &{} . \\ y_{i_1} &{} y_{i_2} &{} ... &{} y_{i_{n_{{\text{items}}}}} \\ . &{} . &{} &{} . \\ . &{} . &{} ... &{} . \\ . &{} . &{} &{} . \end{bmatrix} \end{aligned}$$

and user–item ratings as the interaction matrix ${\hat{R}}$ approximating true *R*

$$\begin{aligned}{\hat{R}} := (\hat{r_{ui}}):= XY^{T}. \end{aligned}$$

Using a simple Boolean variable denoted $p_{ui}$, we want to determine whether a patient *u* has a preference for skill (domain and target codes) *i*.

For each patient, the SRS-2 T-scores is interpreted as rating/confidence in the model. Following the idea of matrix factorization [103, 106, 107], we find an user (patient) vector $x_u$ for each user *u* and an item (skill) vector $y_i$ for each item *i* so that $p_{ui}$ $\sim$ $x^{T}_{u}y_{i}$.

We try to minimize the $L^{2}$ cost function in Eq. 1:

1 $$\begin{aligned} \begin{aligned} C_{{\text{implicit}}}:=\sum _{u,i\in observed \, interactions} c_{ui}(p_{ui} - x_{u}^{T}y_{i})^{2} \\ + \lambda (\sum _{u} \Vert x_{u} \Vert ^{2} + \sum _{i} \Vert y_{i} \Vert ^{2} ), \end{aligned} \end{aligned}$$

where the constant $\lambda$ is the regularization parameter that helps to penalize the large-magnitude components of the matrices *X* and *Y* for numerical stability. The more a patient interacts with a skill, the more we penalize our model for incorrectly predicting $p_{ui}$. If a patient has never interacted with a skill, it is possible that $p_{ui}=1$ and the skill are not part of the treatment plan. To overcome the challenge, we defined the degree of confidence $c_{ui}$ depicted by Equation 2:

2 $$\begin{aligned} c_{ui}:= 1 + \alpha r_{ui}, \end{aligned}$$

where $\alpha$ is a model parameter that must be tuned on our data. There is empirical evidence [103] that the sparsity ratio (the ratio of nonzero entries to zero entries) threshold value serves as a benchmark and that missing entries are frequently regarded as somewhat negative, suggesting that *alpha* balances positive and negative interactions. Our data have a sparsity ratio of 0.94, which is less than the critical value of 0.995, above which model performance declines significantly. The implementation of the implicit Feedback ALS is discussed in detail in Algorithm 2. We used 80% of user and item vectors for the training algorithm and masked 20% of items to blind validate model performance. Our goal is to minimize $C_{{\text{implicit}}}$ by keeping user vectors fixed and solving the quadratic equation for item vectors decreasing $C_{{\text{implicit}}}$. Now, we alternatively keep item vectors fixed and solve the quadratic equation 1 for user vectors until $C_{{\text{implicit}}}$ converges to the global minimum. Table 2 highlights ALS model training parameters. We added a regularization term with a value of 10 to make the trained model less scale-dependent. We can expect similar performance if we apply the best parameter learned from a sampled subset to the entire dataset. We evaluate the model’s recommendation on masked user and item vectors to determine the most appropriate treatment recommendation outcomes (domain/target code) for the user, i.e., the study participant. The number of latent factors that should be recommended for both the domain and target codes was determined using cross-validation. These variables influence the amount of abstract data stored in a two-dimensional space. A matrix factorization based on a single latent component is analogous to a recommender system that automatically recommends the items with the highest number of interactions. Increasing the number of latent factors improves personalization up to a point where the model overfits. We chose 22 and 20 as the optimal latent factor values for domain and target code recommender models, respectively, as specified in Table 2.

**Table 2:** Implicit feedback ALS model training parameters

Parameter	Target code	Domain code
Number of latent factors	20	22
Regularization	0.10	0.10
Iterations	200	150

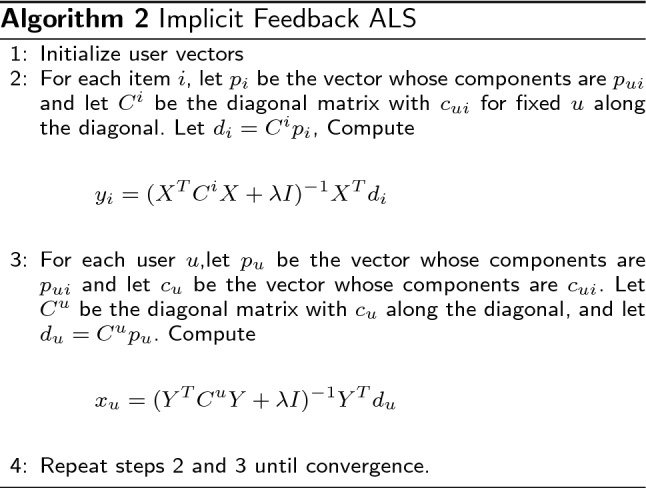



## Results

The results of the commonality and treatment effectiveness measures are presented in Sects. 4.1 and 4.2, respectively. In each subsection, we present the results of two machine learning models: patient similarity and collaborative filtering.

### Commonality measure

This subsection discusses the results of the commonality and other evaluation metrics for patient similarity and the CF machine learning models.

#### Patient similarity

In the first step, we compute each participant’s similarity to the top three other study participants using the cosine similarity [100]. We further compute commonality scores as listed in Table 5 for each participant,and evaluation metrics on five measures in Tables 3 and 4 referring to the top three similar participants for domain and target recommendations, respectively. The five evaluation measures are precision, recall, accuracy, F1 score, and AUC. These measures incorporated computation of true positive (TP), true negative (TN), false positive (FP), and false negative (FN), denoting a recommender system’s outcomes compared to the ground truth. The items for evaluation metrics are defined below.

**Fig. 3:**
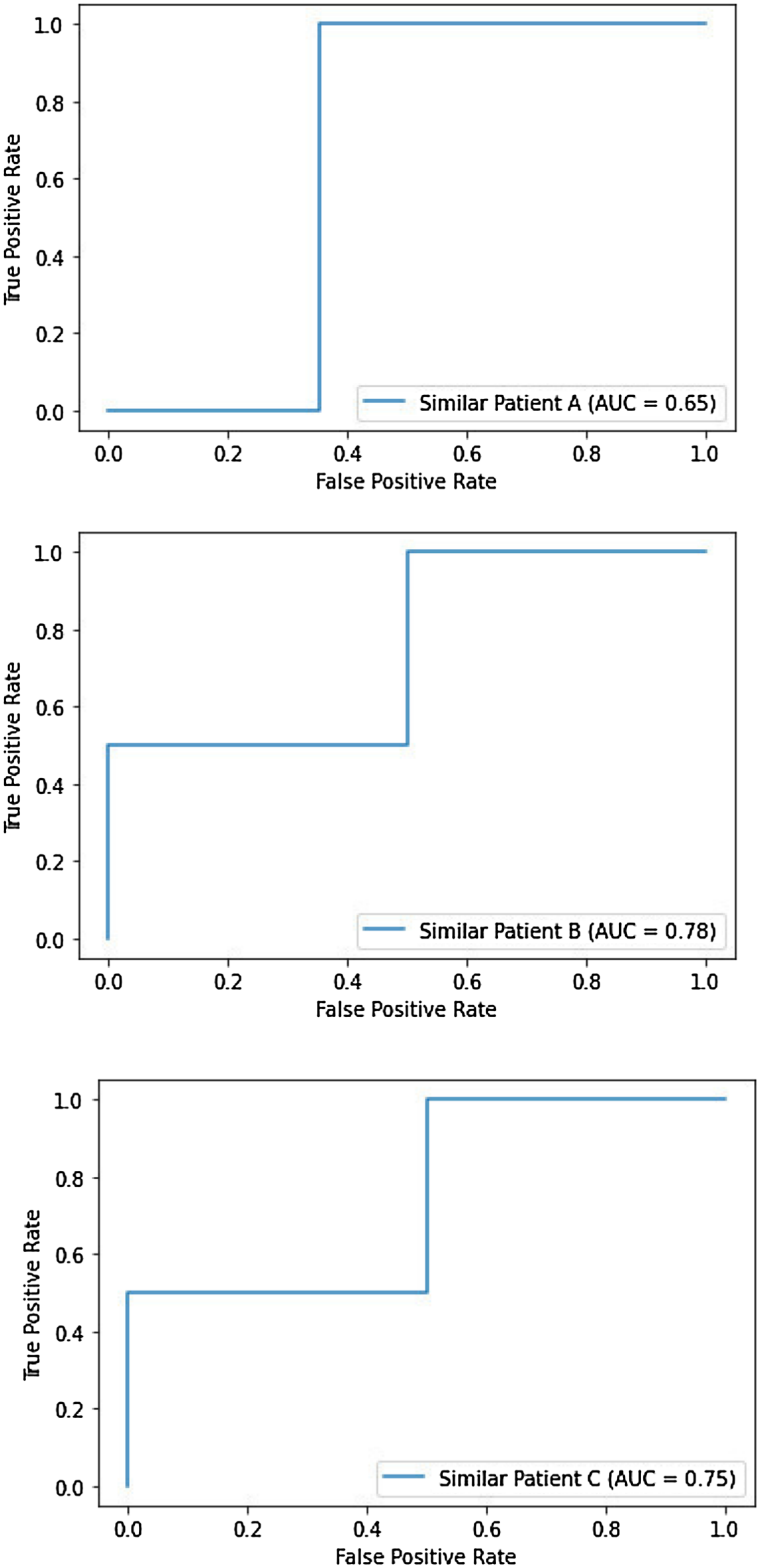
ROC curve for domain recommendations

**Fig. 4:**
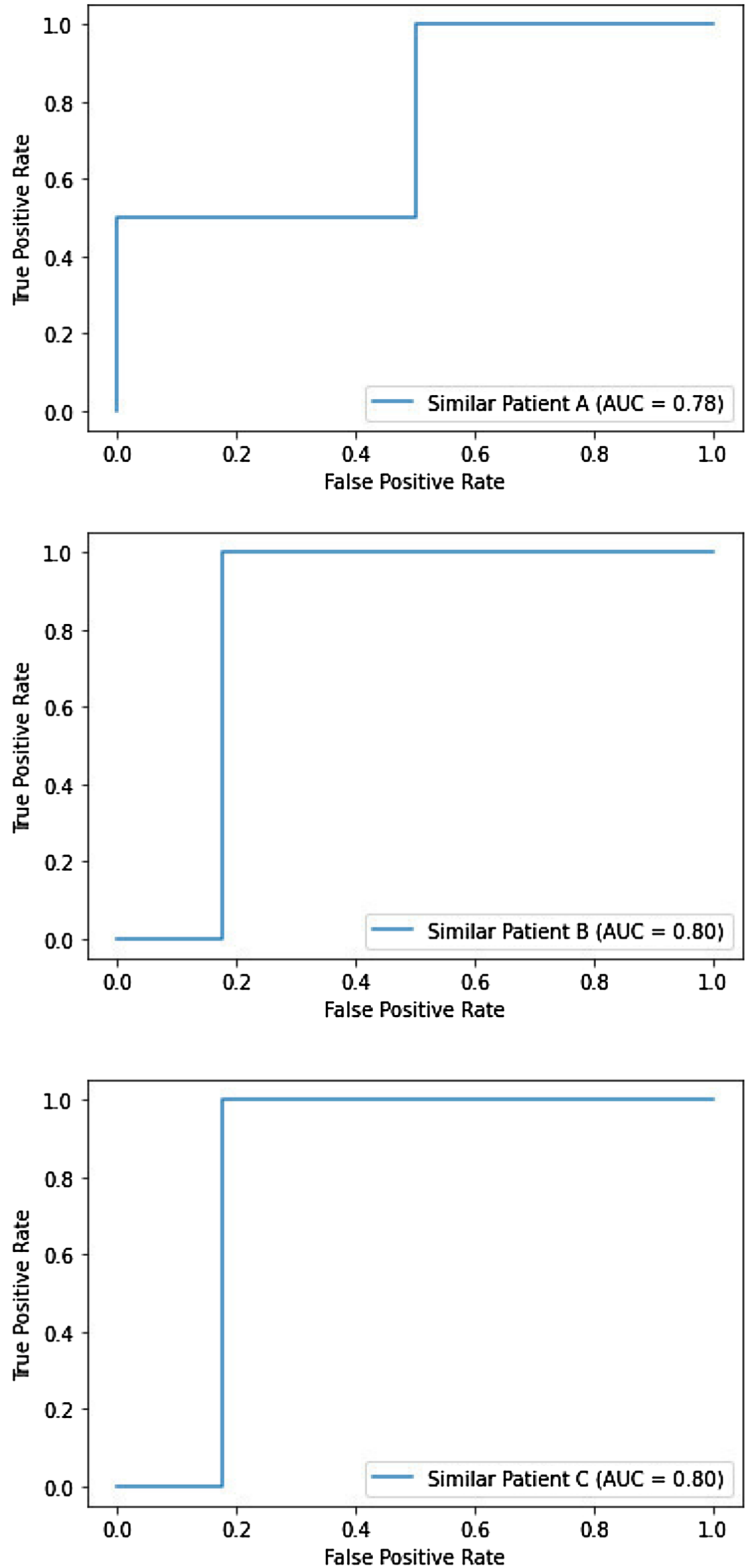
ROC curve for target recommendations

- *Precision* [108] is a ratio of accurately predicted positive recommendations (TP) to all positive recommendations (TP+FP). Precision may be expressed mathematically as the following equation, and the optimal precision value for an ideal recommender system is 1. The average precision outcome for 29 research participants, considering the top 3 similar participants, was 0.64–0.92 for domain recommendations and 0.85–0.90 for target suggestions. Given that targets are a subset of the domain, it is reasonable to predict that the joint probability distribution of target recommendation is dependent on the successful recommendation of domain code as a first step of the treatment plan:
  $$\begin{aligned} {\text{Precision}} =\frac{\text {TP}}{\text {TP} + \text {FP}}. \end{aligned}$$
- *Sensitivity or recall* [108], is computed as the ratio of accurately predicted positive recommendations (TP) to actual positive recommendations (TP+FN) and is denoted by the mathematical formula below. Similar to precision, recommendations with a recall score of one are optimal. It is critical to obtain a true-positive outcome in medical and clinical studies, emphasizing the critical role of evaluation criteria, specifically precision and recall. The higher the precision and recall values, the more robust the recommendation outcome is. The average recall values were near-perfect 1 for the domain level and 0.96 for the target level, suggesting the recommender’s robustness:
  $$\begin{aligned} {\text{Recall}} =\frac{\text {TP}}{\text {TP} + \text {FN}}. \end{aligned}$$
- *Accuracy * [108] is computed as the count of accurate suggestions (TP+TN) divided by the total count of suggestions (TP+TN+FP+FN) and is expressed mathematically below. One disadvantage of the accuracy metric is that it ignores the complexities of class imbalances and the various costs of false negatives and positives. However, accuracy measures suggest how closely the evaluation criteria used by study participants match population characteristics, indicating that the research is generalizable, dependable, and valid. The accuracy outcomes for domain and target recommendations were substantial, ranging between 0.72–0.94 and 0.83–0.87:
  $${\text{Accuracy}} =\frac{{\text{TP}}+{\text{TN}}}{{\text{TP}} + {\text{TN}}+{\text{FP}} + {\text{FN}}}.$$
- *The F1 score* [108] is the harmonic mean of precision and recall. The F1 score is a more robust metric than accuracy since it considers the specific costs of false positives and negative recommendations based on unequally dispersed healthcare class data. A high F1 score suggests that the recommendation system is robust and produces few false positives and negatives. F1 can be represented numerically using the following equation. While the F1 score for domain code suggestions was moderate, ranging between 0.78 and 0.96, we discovered robust treatment recommendations for targets, ranging between 0.90 and 0.93. The results auger well from a clinical perspective as targets are incorporated as a part of the treatment plan to overcome deficits in social communication, academics, prerequisites, and behavior management for study participants:
  $$\begin{aligned} F1 =\frac{\text {2*TP}}{\text {2*TP} + \text {FP}+\text {FN}}. \end{aligned}$$
- *The area under the curve-receiver operating characteristics (AUC-ROC)* [108] score determines the ML model’s robustness. AUC values of 0.5 indicate that recommendations are random, values between 0.6 and 0.8 indicate that recommendations were good, values between 0.8 and 0.9 indicate that recommendations were excellent, and values greater than 0.9 indicate that recommendations were outstanding. ROC curves are frequently employed to illustrate the trade-off between sensitivity and specificity for all conceivable cut-off values in a test. The optimal cut-off has high true-positive and low false-positive rates. The *X*-axis indicates the false-positive rate, and *Y*-axis depicts the true-positive rate. Additionally, AUC scores are crucial for medical research since they provide a relevant interpretation regarding the commonality measure. The AUC values for the domain were modest, ranging between 0.65 and 0.74, but the AUC scores for targets were outstanding, ranging between 0.78 and 0.80. The AUC-ROC curves for the top three patients for domain and target codes are shown in Figs. 3 and 4, respectively.

**Table 3:** Psychometric properties for domain code recommendations

Metric	Similar participant A	Similar participant B	Similar participant C
Precision	0.64	0.71	0.92
Recall	1.0	1.0	1.0
Accuracy	0.72	0.77	0.94
F1 score	0.78	0.83	0.96
AUC score	0.65	0.78	0.74

**Table 4:** Psychometric properties for target code recommendations

Metric	Similar participant A	Similar participant B	Similar participant C
Precision	0.85	0.87	0.90
Recall	0.96	0.96	0.96
Accuracy	0.83	0.84	0.87
F1 score	0.90	0.91	0.93
AUC score	0.78	0.80	0.80

The commonality metric displayed in Table 5 suggests a percentage accuracy of the recommended domain and target for each participant compared to those built by clinicians (ground truth). The findings suggest that all participants recorded a minimum of 65% percent commonality, implying that the recommendations are acceptable.

We observed a broad domain recommendation accuracy range for the participants. The result suggests greater than 90% commonality for six participants, 80–90% for thirteen, 70–80% for seven, and less than 70% for three participants. The domain recommendation metric had a more pronounced frequency distribution than the target recommendation metric.

Similarly, for target recommendations, we observed between 80-90% commonality for 24 participants, greater than 90 and less than 70 for 2 participants each, and between 70 and 80% for one participant. These values suggest that the outcomes of ML models are stable and consistent in their performance. Further, the average commonality score for domain and target codes was 82.86 and 84.07 for all 29 participants.

**Table 5:** Results of patient similarity recommendations for domain and target on commonality measure

Participant	Age in months	Gender	Domain code recommendation	Target code recommendation	Relevant percentage of recommended domain code	Relevant percentage of recommended target code
Participant 1	50.4	Male	2, 4, 5, 14	2.1, 2.3, 4.2, 5.1, 14.1	94.4	80.0
Participant 2	60	Male	1, 2, 4, 5, 6, 7, 11, 14	0.0, 1.1, 2.1, 2.3, 5.1, 5.13, 5.2, 5.8, 6.1, 7.12, 7.3, 11.1, 14.1, 14.3	83.3	66.6
Participant 3	60	Male	1, 2, 4, 7, 14	1.3, 2.1, 2.3, 7.12, 14.1, 14.3	94.4	81.4
Participant 4	72	Male	2, 4, 14, 20	4.2	83.3	85.1
Participant 5	48	Male	1, 2, 4, 7, 14	2.1, 2.3, 7.12, 14.1, 14.3	94.4	82.4
Participant 6	57.6	Male	1, 5	5.4, 9.1	66.6	84.2
Participant 7	66	Female	1, 4, 5, 11	1.1, 4.2, 11.1	77.7	83.3
Participant 8	55.2	Female	2, 4, 5	2.1, 2.3, 5.1	88.8	89.8
Participant 9	31.2	Male	1, 4, 5	1.1, 5.1	83.3	87.0
Participant 10	57.6	Male	1, 2, 4, 5, 17	1.1, 4.2, 5.1	83.3	87.0
Participant 11	45.6	Male	1, 2, 4, 5, 11, 17	1.1, 2.1, 4.2, 5.1, 11.1, 11.2	88.8	81.5
Participant 12	50.4	Male	2, 4, 5, 6, 14	4.2, 5.1, 6.3	95.2	87.0
Participant 13	54	Male	2, 4	2.1, 4.2	66.6	83.3
Participant 14	46.8	Male	1, 5, 11, 20	1.1, 1.3, 5.1, 5.2, 11.1, 11.2	77.7	80.5
Participant 15	49.2	Male	4	1.3	55.5	77.2
Participant 16	49.3	Male	1, 2, 4, 5, 11	1.1, 2.3, 5.1, 11.2	94.4	87.9
Participant 17	33.6	Male	1, 4, 5, 6, 7	0.0	93.3	58.3
Participant 18	62.4	Male	4	1.3, 2.1, 2.3, 7.12	77.7	87.9
Participant 19	45.6	Female	2, 4	4.2, 5.1	72.2	91.6
Participant 20	37.2	Male	1, 2, 4, 5	1.1, 5.1	88.8	97.8
Participant 21	40.8	Male	2, 4, 5, 6	5.1, 6.25	88.8	84.2
Participant 22	48	Male	1, 4, 5	1.1, 5.1	83.3	87.0
Participant 23	27.6	Male	4, 5	5.1	77.7	82.4
Participant 24	55.2	Male	2, 4, 5, 6, 14	4.2, 5.1, 6.3	83.3	82.4
Participant 25	54	Male	1, 4, 5	1.1, 5.1	83.3	87.0
Participant 26	62.4	Male	1, 2, 4, 5	1.1, 2.1, 5.1	88.2	89.8
Participant 27	48	Male	7	7.12	77.7	89.8
Participant 28	26.4	Male	4, 5	5.1	77.7	88.8
Participant 29	56.4	Male	1, 4, 5	1.1, 5.1	83.3	87.0
Average scores	50.03	26 male, 3 female			82.86	84.07

#### Collaborative filtering

Precision, recall, and the F1 score metrics can assess a recommendation system’s robustness [68, 108, 109]. These measures aid clinicians in making sound judgments about the dependability of therapy recommendations and implementing them into treatment plans. However, these evaluation metrics are global and apply to the entire data set, rather than focusing exclusively on the “Top-N” most appropriate recommended items. For example, if a clinician is presented with a list of 100 treatment goals, the first 5, 10, or 20 are the most critical and are most likely to be evaluated by the clinician. As a result, ranking the recommendation system’s results is critical. Therefore, the standard evaluation outcomes have to be constrained top-n bound into rank-aware metrics to increase their precision and recall in the context of a recommendation system. Precision@N and Recall@N can be used to accomplish this with top N recommendations with results comparable to those of precision, recall, and F1 measures. Additionally, top-n bound rank-aware evaluation metrics can prioritize critical items extremely high on the list of recommendations using two distinct metric families approach. The first is a binary classification metric that determines whether a treatment recommendation is appropriate or not. The second metric is utility-based, quantifying recommendation item’s absolute or relative relevance and ranking them according to their appropriateness. We evaluate in Tables 6 and 7 recommendation outcomes of the collaborative filtering model on three evaluation metrics, P@k, MAP@k, and NDCG@k (with* k*=5). The value of* k*=5 resulted in the best psychometric outcomes compared to the outcomes of* k* = 1, 3, 5, 10, and 20.

**Table 6:** Evaluation metrics of domain recommendation

Metric	Value
P@k	0.77
MAP@k	0.75
NDCG@k	0.79

**Table 7:** Evaluation metrics of target recommendation

Metric	Value
P@k	0.85
MAP@k	0.77
NDCG@k	0.81

The items of evaluation metrics are detailed below. We calculated P@K, k=5, by taking the participant’s top five recommendations and counting the number of relevant recommendations matched with ground truth. This number divided by k yields the P@K:

- *Precision@k(P@k)* [110] is proportion of recommended items in the top-k set that are relevant. For domain and target suggestions, we observed robust outcomes of 0.77 and 0.85 for P@5. The accuracy of recommendations was higher for targets than for the domain, which clinically bodes well as targets are part of treatment goals to overcome participants’ skill deficits:
  $$\begin{aligned} {\text{P@k}} =\frac{ \text {Number of recommended items @k that are relevant}}{\text{ Number } \text{ of } \text{ recommended } \text{ items } \text{@k }}. \end{aligned}$$
- *Average Precision@k (AP@k)* is the number of top-k relevant items recommended of *m* total items [111]. AP@K is calculated for a single user. To find AP@K, for example, we added P@1, P@2,..., and P@K and divided that by k:
  $$\begin{aligned} {\text{AP@k}} = {\frac{1}{m}} \sum _{k=1}^{N} P(k)\cdot rel(k) , \end{aligned}$$
  where *rel*(*k*) indicates whether $k$th item was relevant or not.
- *Mean Average Precision@k(MAP@k)* is the average of AP@k over all users *U* [112] and can be represented with the following equation:
  $$\begin{aligned} {\text{MAP@k}} = {\frac{1}{|U|}} \sum _{u=1}^{N} {\text{AP@k}}. \end{aligned}$$
  To calculate MAP@k, we added the AP@k values for all users and divided them by the number of study participants (k). For domains and target recommendations, the MAP@5 results were almost similar, with values of 0.75 and 0.77, respectively.
- *Cumulative Gain@k (CG@k)* is the sum of the gains associated with the first k recommended items in any sequence. Gain is the score assigned to each recommended item based on its relevancy, and CG is the sum of all recommendation outcomes graded relevance scores [113]. The challenge with CG is that it ignores the result set’s rank when calculating its utility:
  $$\begin{aligned} {\text{CG@k}} = \sum _{i=1}^{K} G_i. \end{aligned}$$
- *Discounted Cumulative Gain@k (DCG@k)* weighs each recommendation score based on its position. For example, the top items in the recommendation are rated higher, and the bottom items with a lower score. DCG penalizes highly relevant recommendations that appear lower in the search result list by decreasing the graded relevance value logarithmically proportional to the position of the recommendation in the result [113]:
  $$\begin{aligned} {\text{DCG@k}} = \sum _{i=1}^{K} \frac{G_i}{log_2(i+1)}. \end{aligned}$$
- *Normalized Discounted Cumulative Gain@k (NDCG@k)* is the DCG@k over a normalization factor. It evaluates both the degree of relevance and the ranking of items in recommendations. As the length of the recommendation outcomes varies based on input parameters, the NDCG is calculated by normalizing the cumulative gain at every item’s recommendation position [113]:
  $$\begin{aligned} {\text{NDCG@k}} = {\frac{{\text{DCG@k}}}{{\text{IDCG@k}}}}, \end{aligned}$$
  where IDCG@k denotes the Ideal DCG when the system recommends the most relevant items first:
  $$\begin{aligned}{\text{IDCG@k}} = \sum _{i=1}^{K^{{\text{ideal}}}} \frac{G_i^{{\text{ideal}}}}{log_2(i+1)}. \end{aligned}$$
  The real challenge with NDCG is that, when only partial relevance feedback is available, we typically do not know the ideal ordering of results. The NDCG, on the other hand, has been demonstrated to be a valuable statistic for measuring the quality of ranking for a range of problems, including job offer [114], BBC news [115], and Airbnb bookings [116] recommendations. We observed the NDCG@5 score highest among the three evaluation parameters. The recommendation ranged between 0.79 and 0.81 for NDCG@5 for domain and target, respectively.

When compared to the ground truth, the commonality measure shown in Table 8 indicates a percentage of accuracy for a participant-level treatment recommendation for domain and targets.

We observed a broader domain recommendation scores on commonality measure of more than 90% for three participants, 80–90% for 14 participants, between 70 and 80% for 11 participants, and less than 70% for one participant, implying that the recommendations are robust.

Similarly, for target recommendations on commonality measure, we observed between 80 and 90% score for 20 participants, greater than 90% but less than 70% for none, and between 70 and 80% for nine participants. These values imply that the output of machine learning models is stable and consistent in its performance. The average commonality scores for all 29 participants for domain and target recommendations were 81.8 and 82.32, respectively. The domain recommendation measure demonstrated a broader frequency distribution than the target recommendation on commonality measure.

**Table 8:** Results of domain and target recommendation of all participants using collaborative filtering model on commonality measure

Participant	Age in months	Gender	Domain code recommendation	Target code recommendation	Percentage of relevant domain code	Percentage of relevant target code
Participant 1	50.4	Male	1, 2, 3, 4, 5, 14	2.1, 2.3, 2.4, 3.2, 3.4, 14.3	87.5	88.3
Participant 2	60	Male	2, 4, 5, 6	2.1, 2.2, 2.3, 2.4, 5.1, 5.13, 5.2, 5.8, 6.1, 6.11, 6.14, 11.1, 14.1, 14.3	88.0	77.6
Participant 3	60	Male	1, 2, 4, 7	1.1, 1.2, 1.3, 2.1, 2.4, 2.3, 7.12, 7.19	89.2	87.3
Participant 4	72	Male	2, 4, 14, 20, 21	2.2, 2.3, 2.4, 4.2, 4.7, 20.3, 21.02	77.5	78.9
Participant 5	48	Male	1, 2, 4, 14	1.5, 1.6, 1.7, 2.1, 2.3, 4.1, 4.2, 14.1, 14.3	94.4	82.4
Participant 6	57.6	Male	1, 5, 6	1.3, 5.4, 6.2	73.0	88.4
Participant 7	66	Female	4, 5, 11	4.1, 4.2, 11.1, 11.2, 11.3	73.7	79.4
Participant 8	55.2	Female	2, 4, 5	2.1, 2.2, 4.2, 4.5, 5.1, 5.4	91.2	86.4
Participant 9	31.2	Male	4	4.1, 4.2, 4.4	72.3	81.0
Participant 10	57.6	Male	1, 4, 5, 17	1.1, 1.2, 4.2, 4.3, 4.4, 5.1, 5.2, 17.2	84.2	87.2
Participant 11	45.6	Male	1, 2, 4, 5, 11	1.1, 2.1, 4.2, 5.1, 5.4, 11.1, 11.2	85.9	77.4
Participant 12	50.4	Male	2, 4, 6	2.1, 4.2, 6.21, 6.3, 6.6	95.2	87.0
Participant 13	54	Male	2, 4	2.1, 4.1, 4.2	70.4	88.8
Participant 14	46.8	Male	1, 5, 11, 20	1.1, 1.3, 5.1, 5.2, 11.1, 11.2, 11.3, 20.1, 20.5	75.5	82.4
Participant 15	49.2	Male	4, 9	4.2, 4.3, 9.1, 9.2, 9.4	77.1	76.4
Participant 16	49.3	Male	1, 2, 4, 5	1.1, 1.3, 2.1, 2.3, 5.1, 5.2	88.2	83.6
Participant 17	33.6	Male	1, 4, 6	1.2, 1.4, 1.5, 4.1, 4.5, 6.1	86.0	74.4
Participant 18	62.4	Male	2, 4	2.1, 2.3, 2.4, 4.1, 4.3, 4.5	82.5	84.3
Participant 19	45.6	Female	2, 4, 6	2.2, 2.3, 4.1, 4.2, 6.2	76.6	71.0
Participant 20	37.2	Male	1, 2, 4, 5	1.1, 1.2, 4.1, 5.1	85.5	88.7
Participant 21	40.8	Male	1, 2, 4, 5, 6	1.2, 1.4, 1.5, 4.2, 5.1, 6.22, 6.25	83.1	85.6
Participant 22	48	Male	1, 4, 5, 7	1.2, 1.3, 4.2, 4.4, 5.1, 5.2, 7.1	77.3	82.4
Participant 23	27.6	Male	3, 4, 5	3.1, 4.2, 4.4, 5.1, 5.2	73.7	85.8
Participant 24	55.2	Male	1, 2, 4, 5, 6, 14	1.2, 1.3, 1.5, 1.6, 2.2, 2.3, 4.2, 4.7, 5.1, 5.4, 6.1	79.4	88.2
Participant 25	54	Male	1, 4, 5	1.3, 4.1, 4.2, 5.2, 5.4	88.5	72.4
Participant 26	62.4	Male	1, 2, 4	1.1, 2.1, 2.3, 4.3, 4.4	80.7	82.5
Participant 27	48	Male	7, 14	7.1, 14.2	68.3	75.2
Participant 28	26.4	Male	1, 4, 5	1.2, 1.4, 4.1, 4.3, 5.1, 5.2	83.4	81.0
Participant 29	56.4	Male	1, 2, 4, 5	1.1, 1.3, 2.2, 2.4, 5.1, 5.2	85.5	83.5
Average scores	50.03	26 Male, 3 Female			81.85	82.32

### Effectiveness measure

Additionally, we computed recommendation outcomes on efficacy measures using both patient similarity and CF models using data from months 1–3, 4–6, and 1–6. Treatment effectiveness (TE) results are listed in Table 9 and suggest how many of the participant’s recommended targets were mastered or acquired by the participant.

For months 1–3, 4–6, and 1–6, the patient similarity model’s domain code recommendations for TE measure were 82.1%, 85.31%, and 84.0%. The results for CF in the same period were 74.82%, 62.06%, and 76.89% points.

Similarly, for months 1–3, 4–6, and 1–6, the patient similarity model’s domain code recommendations for TE measure were 90.68%, 89.96%, and 90.34%. The TE results for CF in the same period were 65.51%, 55.51%, and 58.27% points, significantly lower than the patient similarity model.

The experiment results demonstrate that recommendations for patient similarity and CF models are comparable on commonality measures. However, the patient similarity model outperformed the CF model on effectiveness measures.

There are a few reasons for the poor outcomes shown by the CF model. Firstly, the CF incorporates a feature vector or embedding of each participant and their clinical meta-data, including assessment records and treatment histories. However, at the start of the treatment recommendation, no treatment history is available to the algorithm for analysis. This is a typical cold start challenge as CF algorithms generate suggestions based on the item’s interactions that build over time. Therefore, as participant-level recommendations are generated based on item interactions, the CF will unlikely recommend an item during the cold-start stage because of the sparsity and limited user base in the recommendation matrix. The limitation would lead to the CF model’s inability to find similarities between the two participants, rendering CF recommendations ineffective.

Secondly, compared to the patient similarity model, the CF and other matrix factorization models utilize latent features in latent space, complicating recommendation selection. However, treatment recommendation for participants based on their similarity is typically denoted by a dot product number, with a higher value indicating a higher degree of similarity.

Thirdly, whereas the patient similarity model considers the degree of similarity between two participants, the CF model also considers item interactions at the treatment effectiveness level among participants. The treatment efficacy interactions are constructed temporally, have unique trajectories for each participant, and are thus bound by the sparsity ratio, meaning that there is insufficient interaction between participants and assessment features to make an effective recommendation.

Further, CF, compared to the patient similarity model, has computational challenges and performance issues. Due to the complexity of latent sparse matrix optimization, computations are slow at retraining new data in terms of performance. In comparison, retraining a patient similarity model takes only a few minutes.

**Table 9:** Month-wise recommendations accuracy of mastered domains and targets on effectiveness measure

Participant	Age in months	Gender	Similarity model						Collaborative filtering model					
			Months 1–3		Months 4–6		Months 1–6		Months 1–3		Months 4–6		Months 1–6	
			Domain code	Target code	Domain code	Target code	Domain code	Target code	Domain code	Target code	Domain code	Target code	Domain code	Target code
Participant 1	50.4	Male	88	98	94	91	82	93	80	40	80	50	80	80
Participant 2	60	Male	88	87	94	89	82	84	60	60	60	60	80	60
Participant 3	60	Male	71	96	94	92	76	92	60	40	100	60	80	40
Participant 4	72	Male	71	90	94	96	76	90	60	60	80	40	80	80
Participant 5	48	Male	100	78	94	90	94	91	100	100	60	80	60	50
Participant 6	57.6	Male	100	99	94	96	94	89	80	50	80	60	60	50
Participant 7	66	Female	88	87	94	93	100	91	60	60	60	40	80	40
Participant 8	55.2	Female	88	97	94	91	100	92	100	100	100	80	100	80
Participant 9	31.2	Male	65	98	71	96	53	99	80	80	80	60	100	80
Participant 10	57.6	Male	65	89	71	90	53	60	80	60	100	100	60	60
Participant 11	45.6	Male	94	92	53	99	82	82	80	80	60	80	80	40
Participant 12	50.4	Male	94	96	53	60	82	92	100	40	40	40	80	40
Participant 13	54	Male	71	99	94	87	94	93	60	60	50	50	60	40
Participant 14	46.8	Male	71	60	94	99	94	91	40	40	40	60	40	40
Participant 15	49.2	Male	94	93	88	87	88	92	70	60	40	40	80	50
Participant 16	49.3	Male	94	95	88	91	88	90	100	100	80	60	100	50
Participant 17	33.6	Male	71	91	65	98	88	87	100	100	40	50	40	60
Participant 18	62.4	Male	71	91	65	89	88	91	60	60	60	50	100	60
Participant 19	45.6	Female	88	87	94	85	76	99	80	40	40	40	50	50
Participant 20	37.2	Male	88	91	94	88	88	90	60	80	80	60	80	60
Participant 21	40.8	Male	76	92	88	83	94	92	60	60	60	40	40	40
Participant 22	48	Male	76	90	88	93	94	96	100	100	60	40	100	80
Participant 23	27.6	Male	76	88	88	93	82	97	60	50	40	40	100	80
Participant 24	55.2	Male	76	92	88	81	82	87	80	50	100	60	80	40
Participant 25	54	Male	94	87	100	91	94	90	80	40	40	60	80	60
Participant 26	62.4	Male	94	99	100	92	94	96	80	50	40	50	80	60
Participant 27	48	Male	82	93	82	93	71	91	60	100	40	60	100	100
Participant 28	26.4	Male	82	84	82	84	76	91	80	100	40	40	100	40
Participant 29	56.4	Male	65	91	76	92	71	92	60	40	50	60	60	80
Avg. Score	50.3	26M,3F	82.1	90.68	85.31	89.96	84	90.34	74.82	65.51	62.06	55.51	76.89	58.27

## Discussion

Over 6 months of treatment data, we built and validated two machine learning algorithms, patient similarity and collaborative filtering to recommend and personalize ABA treatment. The model of patient similarity was trained using sociodemographic data from the participants’ ages, genders, and clinical evaluation records. In addition, the CF model was trained using age, gender, assessment records, treatment history, and effectiveness data. On commonality and effectiveness criteria, the recommended treatment goals of the two machine learning models were compared to those included by clinicians (ground truth) in the treatment plan.

### Patient similarity model

The patient similarity model recommended the three most similar patients for each study participant. We then matched that participant’s treatment record to those of three similar patients. on commonality and five other metrics, i.e., precision, recall, accuracy, F1 score, and AUROC for domain and target codes recommendations as specified in Algorithm 1. Thus, we assess each participant’s relevance to a treatment recommendation inherently captured by sociodemographic data (age and gender of participants), SRS-2, and VB-MAPP assessment scores. Tables 3 and 4 demonstrate the robustness of five psychometric measures for recommending domain and target codes. For the top three similar patients, the outcome of five psychometric measures varied from 0.74 to 1, with metrics for target recommendation outperforming that of the domain. Additionally, Table 5 shows that participant-level commonality accuracy metrics averaged 82.8 and 84.07% for domain and target recommendation, respectively. Further on effectiveness measures, the mean TE accuracy of domain recommendation for months 1–3, 4–6, and 1–6 is listed in Table 9 and ranged from 82.1%, 85.31%, and 84% accuracy points. The target-level accuracy metric was 90.68%, 89.96%, and 90.34% points in the same period, exceeding domain-level accuracy measurements.

### Collaborative filtering model

We calculated commonality and three other metrics on the top five CF model recommendations for domain and target codes per the logic specified in Algorithm 2. Thus, we assess each participant’s relevance to a treatment recommendation inherently captured by sociodemographic data, treatment history and effectiveness, and SRS-2 and VB-MAPP assessment scores. Tables 6 and 7 demonstrate the robust evaluation results of treatment recommendations for domain and target codes on evaluation metrics consisting of P@5, MAP@5, and NDCG@5 measures. On commonality measures for the top five treatment suggestions, the outcomes of evaluation metrics ranged between 0.75 and 0.79 for the domain (Table 6) and 0.77–0.85 for the target recommendation (Table 7). Like the patient similarity model, the outcome metrics of target recommendation outperformed that of domain’s. The mean accuracy commonality measure for the participant-level metrics in Table 8 ranged between 81.85 and 82.32% for domain and target code, respectively. However, on the effectiveness measure, the CF model performed poorly with results mentioned in Table 9. The mean accuracy of domain recommendation for months 1–3, 4–6, and 1–6 ranged from 74.82, 62.06%, and 76.89%. The target-level accuracy metric was 65.51, 55.51, and 58.27% points in the same period, performing poorly than the domain-level accuracy measurements.

On commonality measures, the evaluation metrics for precision, recall, and accuracy scores are comparable at participant and aggregate levels, with the patient similarity model outperforming the CF model slightly. Further on commonality measure, the target recommendations metrics outperformed domain recommendations for patient similarity and the CF recommendation model. The reason can be attributed to the low volume and wide variety of data for training ML models resulting in poor recommendations. However, on the effectiveness measure, the patient similarity model outperformed the CF model majorly because of three reasons:

1. Cold start with no treatment effectiveness information at the start of the treatment available for the CF model. However, the patient similarity model can effectively generate the first treatment prescription.
2. Limited treatment and effectiveness records at the initial treatment stages limit the CF model’s performance.
3. A sparse multi-dimension matrix generated from user-level interaction with treatment records for training the CF model limits its performance.

### Comparison of recommendation models

Many real-world data sets are 99 percent (or even more) sparse and have been used to generate robust recommendations. The matrix factorization model simplifies user–item ratings by transforming them into the product of two smaller matrices. One is for users, while the other is for products. In our case, CF aims to recommend treatment based on a user’s prior treatment records and their effectiveness. Using the matrix factorization approach, when we factor a* M* $\times$* N* matrix into two* M* $\times$* K* and* K* $\times$* N* matrices, we reduce “*n*” items to “*k*” factors.

As for item–item interaction, the objective is to predict or prescribe treatment based on the efficacy of similar ABA treatments consisting of domains or targets. Therefore, instead of many treatment records in the system, say 25000, we can have those treatments distributed over 22 domains (verbal operants), each of which has a linear combination with each treatment line item. Thus, a domain may refer to manding, tacting, visual perception, academic language, and social skills and will usually have a relationship with the treatment record. The critical point is that recommending based on factors is more robust than comparing individual patients and their treatments. For example, a user may not have been assigned an ABA treatment goal within the manding or tacting domain but may have other treatment goals related to the tacting domain via some latent factors. Therefore, the factors are latent because they exist in our data, but are not detected until the reduced rank matrix factorization makes those factors emerge. The CF confronts somewhat the issue of cold start due to its reliance on feedback or activity from other users when it has a large user base, even with sparse matrix user interactions. It recommends a treatment ‘x’ to user ‘a’ based on user ‘b”s treatment path and effectiveness. The users ‘a’ and ‘b’ must have previously received similar treatment or may have similar assessments and sociodemographic records, so they are clustered together and form a recommendation basis.

However, item-based recommendations require some historical data to incorporate an implicit feedback loop. Large-item interactions are prohibitively expensive and time-intensive. A small user data volume consisting of a sparse treatment effectiveness matrix with limited interactions may yield modest treatment recommendations.

Further, a collaborative filtering system’s primary objective is to overcome the drawbacks of patient similarity. The CF recommendations consider all users and group them according to their similarities and latent factor associations rather than focusing on a single user. Therefore, rather than combining outcomes of multiple recommendation engines into one, another method is to deliver concurrent recommendations, for example, from patient similarity and CF, and allow the clinician to choose between them. A comparison of patient similarity and CF model is listed in Table 10. The proposed solution may contribute to a pleasant user experience if appropriate explanations are shared to assist the clinician in appreciating the rationale behind the recommendations and their robust psychometric properties.

#### Clinical relevance

ASD is a developmental disorder that affects around 1 in every 44 individuals. The demand for evidence-based interventions such as ABA has outstripped the supply of qualified and licensed clinicians, resulting in a decline in quality, accessibility, and affordability. The findings of this study can increase clinician capacity, allowing them to manage a more significant ASD population, and improve treatment quality through the use of an integrated treatment recommendation and personalized decision support system.

We assume that an EMR application captures multiple information: Firstly, sociodemographic participant information (age and gender); secondly, diagnostic assessment records, for example, from CARS-2 [117], ADI-R [118], ADOS-2 [119]; thirdly, functional assessments records, for example, from VSMS [120], VABS [121], and SRS-2 , and lastly longitudinal treatment records and its effectiveness.

These data points can identify a child’s strengths, skill gaps, and potential improvement areas in social communication, motivation, cognition, motor skills, restricted interests, and repetitive behavior and assist clinicians in developing a personalized treatment plan. However, the design and development of ABA treatment are challenging. Children with ASD typically exhibit a wide range of challenges, and the disorder affects males four times more than females. Further, sociodemographic characteristics such as age, gender, place of residence, access to healthcare, family income, and educational background can affect the treatment design and delivery. The above challenges can be overcome by designing a feature vector during the patient intake to capture diagnostic and functional assessment scores, age, gender, and other sociodemographic characteristics. At the intake stage, using the feature vector, the patient similarity model can compare incoming patients to an extensive patient database to recommend the most similar patients and correlate their treatment trajectory with outcomes, allowing physicians to select the ideal treatment strategy. This can solve the cold-start challenge with no treatment data availability during the treatment initiation stage.

During the intervention steady-stage, the CF model with a feature vector capturing patient treatment records, latent factors, and their effectiveness can recommend and personalize the treatment trajectory based on the treatment outcomes of similar patients.

Therefore, patient initial diagnostic and age, gender, treatment records, treatment effectiveness, and functional assessment records at various temporal data points can pave the way for treatment recommendation and personalization using patient similarity and the CF recommendation model. In addition, we addressed the limitations identified in [41], as our results apply to individuals under the age of six. In contrast, most studies do not include this age group, and we employed data from the participants, only from low- and middle-income country i.e. India.

### Limitations

The study’s primary limitation is the small sample size of 29 participants. The reason for limited data and participant availability can be attributed to only 2% of the population being diagnosed with ASD. Further, the study was executed online at the peak of COVID-19. The participants were recruited if they fulfilled the inclusion criteria and shared the ASD diagnosis report. Therefore, we may not have been able to include a diverse sample of participants with a wide range of baseline ASD severity level.

Further, we compared the patient similarity model recommendations with the top three most similar study participants; however, for CF recommendations, all 29 participants’ top five recommendations were included in calculating psychometric properties. This could be one of the additional reasons for the poor performance of the CF recommendation model when compared to patient similarity, in addition to the cold start challenge. The performance of these models, particularly CF, could be improved by recruiting additional participants who may have extensive interactions with treatment records and their effectiveness.

Further, as SRS-2 and VB-MAPP are manually performed assessments by clinicians, we expected a certain degree of human subjectivity in performing assessments and, therefore, impact the two models’ recommendation outcomes.

Scalability: As the number of users increases, the CF model becomes less scalable. For example, If we have ten million patients and one hundred thousand treatment items, we must create a sparse matrix with a trillion elements.

Further, we have used only the cosine similarity metric in the study. A single distance metric for computing similarity and subsequent recommendations system may result in a biased recommendation. Future studies can incorporate additional similarity metrics to improve the generalizability of the recommendation system.

### Future direction

In future studies, we must perform the following steps:

1. Include additional machine learning recommendation models than patient similarity, and CF.
2. Include a broader range of assessments in addition to SRS-2 and VB-MAPP.
3. Include diagnostic scores as a vector item for recommendation engine training.
4. Include other similarity metrics than cosine.
5. Include other mental health conditions than autism.
6. Baseline the ASD diagnosis for all participants using the same diagnostic tools.
7. The selection of the corresponding treatment plan by ML algorithm is more objective than through the subjective experience of conventional clinicians; yet, the subjective experience of conventional clinicians may be more effective in the therapy. Therefore, future studies must evaluate the degree to which ML-selected outcomes reflect clinician’s goals, the degree to which they differ, and the significance of this difference.

**Table 10:** Comparison of patient similarity and collaborative filtering model

Parameter	Patient similarity model	Collaborative filtering model
Quality of recommendations	Similar	Similar
Retraining	Easy	Difficult
Recommendation explainability	Simple	Complex
Performance constraint	No	Sparsity ratio

## Conclusion

We evaluated the outcomes of two machine learning models using sociodemographic, assessment records, and treatment effectiveness data. This study highlights that machine learning models can predict ABA treatment programs for children with ASD with robust evaluation metrics and augment the capacity of the ABA clinicians. Our experimental findings suggest that by assigning goal prediction and personalization, we may be able to aid in the prioritization of scarce healthcare resources in the management of ASD. We conclude that healthcare systems should investigate the use of predictive models from the diagnostic to intervention stage to recommend and personalize ABA treatment and optimize healthcare resource prioritization and patient care. The results suggest that the patient similarity model during treatment intake can recommend initial treatment goals with 80-85% accuracy compared to ground truth. Further, CF models can learn from treatment records and treatment effectiveness data during the steady treatment stage to personalize the treatment recommendations during various treatment points. Our multi-model personalized recommendation algorithms provide clinicians with enhanced capacity to serve ASD children with personalized therapy recommendations.

## Data Availability

The datasets generated during and/or analyzed during the current study are available from the corresponding author on reasonable request.

## Appendix 1

**SRS-2: Social Responsiveness Scale 2**: The SRS-2 [94] is a user respondent-reported questionnaire that assesses the extent of social deficits and highlights associated ASD symptoms. The assessment takes only 15–20 min to complete and can cater to a population of broad age ranges, enabling it to track symptoms and skill deficits across the lifespan. Teachers, parents, and professionals can use the tool to rate symptoms using a numerical scale that depicts the severity spectrum. Along with a total score indicating the degree of social deficits in individuals with ASD, the SRS-2 gives scores for five Treatment subscales:

1. Social awareness,
2. Social cognition
3. Social communication
4. Social motivation, and
5. Restricted interests and repetitive behavior.

The SRS-2 results and T-scores are standardized by comparing a large sample population’s age, gender, and characteristics.

**VB-MAPP: Verbal Behavior Milestones Assessment and Placement Program**: The VB-MAPP [95] is an assessment, curriculum guide, and skill tracking tool created for children with ASD and language difficulties. The VB-MAPP comprises five components that allow: (1) capturing a baseline performance; (2) providing an intervention direction; (3) monitoring skill acquisition; (4) tracking outcomes, and (5) a framework for curriculum design. Each VB-MAPP skill is quantifiable, developmentally appropriate, and balanced across verbal operants and related skills. These are the details of VB-MAPP five main components:

1. VB-MAPP Milestones Assessment—assesses a child’s existing verbal and related skills and includes 170 measurable learning and language milestones sequentially and proportionately distributed across three developmental levels (0–18 months, 18–30 months, and 30–48 months).
2. VB-MAPP Barriers Assessment—assesses children with autism or other developmental disabilities on 24 common learning and language acquisition barriers such as behavioral issues, instructional control, defective commands, defective scanning, defective conditional discriminations, and failure to generalize.
3. The VB-MAPP Transition Assessment consists of 18 assessment areas that provide a quantifiable basis for a child’s IEP development, enabling the team to make decisions and establish priorities regarding the child’s educational needs.
4. Task Analysis and Supporting Skills—the VB-MAPP Protocol includes task Analysis and supporting Skills for 14 of the Milestones Assessment’s 16 domains. The supporting skills complement the milestones by emphasizing the importance of developing critical language, learning, and social skills concurrently with the milestones.
5. VB-MAPP Placement and IEP Goals—corroborate the four assessments above. The placement guide provides detailed guidance for 170 milestones and suggestions for IEP goals.

## Abbreviations

ABA: Applied behavior analysis
ADHD: Attention deficit hyperactivity disorder
ADI-R: Autism diagnostic interview-revised
ADIS: Anxiety Disorders Interview Schedule
ADOS-2: Autism Diagnostic Observation Schedule-2
ALS: Alternating least square
AP@k: Average precision at k
ASD: Autism spectrum disorder
AUC: Area under the ROC curve
BCBA: Board certified behavior analyst
CARS-2: Childhood Autism Rating Scale-2
CDS: Clinical decision support
CF: Collaborative filtering
CHR: Clinical high-risk
C3I: Computer-mediated caregiver–child interaction
CNN: Convolutional neural network
CG@k: Cumulative gain at k
DCG@k: Discounted cumulative gain at k
DL: Deep learning
DSM-V: Diagnostic and statistical manual of mental disorders-5
DTR: Dynamic treatment regime
EEG: Electroencephalogram
EIBI: Early Intensive Behavioural Intervention
EMR: Electronic medical record
FN: False negative
fMRI: Functional magnetic resonance imaging
FP: False positive
FPR: False positive rate
GDD: Global development delay
HR-ASD: High-risk autism spectrum disorder
IDCG@k: Ideal discounted cumulative gain at k
IEP: Individualized education program
IJA: Initiating joint attention
INDT-ASD: INCLEN diagnostic tool for autism spectrum disorder
ISAA: Indian scale for identification of autism
LSML: Locally supervised metric learning
MAP@k: Mean average precision at k
ML: Machine learning
NDCG: Normalized discounted cumulative gain
PSM: Patient similarity metric
P@k: Precision at k
RNN: Recurrent neural network
ROC: Receiver operating characteristic
RS: Recommendation systems
SL: Supervised learning
SRS-2: Social Responsiveness Scale-2
T-scores: Total scores
TE: Treatment effectiveness
TN: True negative
TP: True positive
TPR: True-positive rate
VABS: Vineland adaptive behavior scales
VB-MAPP: Verbal Behavior Milestones Assessment and Placement Program
VSMS: Vineland Social Maturity Scale
